# Bradycardia, Renal Failure, Atrioventricular Block, Shock, and Hyperkalemia (BRASH) Syndrome Emergence in a Unique Intersection of COVID-19 and End-Stage Renal Disease: A Case Report

**DOI:** 10.7759/cureus.54695

**Published:** 2024-02-22

**Authors:** Tutul Chowdhury, Sindhu C Pokhriyal, Uma Gupta, Kalendra Kunwar, Kiran Hashmi, Sauraj Devkota, Morris Kopyt, Andleeb Sherazi

**Affiliations:** 1 Internal Medicine, One Brooklyn Health-Interfaith Medical Center, Brooklyn, USA; 2 Internal Medicine, One Brooklyn Health-Brookdale University Hospital Medical Center, Brooklyn, USA; 3 Critical Care, One Brooklyn Health-Interfaith Medical Center, Brooklyn, USA

**Keywords:** beta blockers, hyperkalemia, bradycardia, continuous renal replacement therapy (crrt), pacemaker, heart block, covid-19 infection, end stage renal disease, brash syndrome

## Abstract

Bradycardia, renal failure, atrioventricular (AV) block, shock, and hyperkalemia (BRASH) syndrome is a rare clinical entity that poses challenges for healthcare practitioners. It is characterized by bradycardia, renal failure, atrioventricular (AV) obstruction, shock, and hyperkalemia. This case is an interesting instance of BRASH syndrome in the setting of COVID-19 infection and end-stage renal disease (ESRD). Initial laboratory results revealed macrocytic anemia, renal dysfunction, acidosis, and mild hyponatremia, along with hyperkalemia. An electrocardiogram (EKG) and telemonitoring showed dopamine-resistant persistent bradycardia until transvenous temporary pacemaker placement was done, which resolved the bradycardia. Anti-hyperkalemic therapy, avoiding AV nodal-blocking medication, and temporary pacemaker placement were all part of the management. After receiving hemodialysis, the patient gradually recovered. Bradycardia improved and potassium normalized. The intricate interaction between hyperkalemia and AV nodal obstruction that causes BRASH syndrome results in severe bradycardia and shock. To the best of our knowledge, this is the first case of BRASH syndrome in a patient with an active COVID-19 infection in a previously vaccinated patient. Even though case reports make up the majority of the material currently in publication, to fully comprehend the mechanisms underlying this illness, more research is required, as early detection of this syndrome is crucial for better patient outcomes.

## Introduction

Bradycardia, renal failure, atrioventricular (AV) block, shock, and hyperkalemia (BRASH) syndrome is a unique and potentially fatal condition characterized by its simultaneous occurrence. Its pathogenesis is a complicated combination of renal and cardiac failure that can quickly escalate to hemodynamic instability [1,2]. Here, we present a case of BRASH syndrome in a patient with a COVID-19 infection and pre-existing ESRD. While respiratory distress and pneumonia have been at the forefront of clinical attention in COVID-19 patients, it has also become increasingly evident that COVID-19 can affect multiple organ systems, leading to a wide array of clinical manifestations. One such manifestation that has emerged as an intriguing and potentially life-threatening complication is bradycardia. However, this case not only had bradycardia but also showed a constellation of signs that met the criteria for BRASH syndrome. The diagnosis of bradycardia associated with this syndrome has significant clinical implications because it can present with a more severe manifestation than originally anticipated and can show resistance to standard bradycardia treatment regimens like this case, which can lead to hemodynamic instability and poor patient outcomes. The majority of people who suffer from BRASH syndrome usually respond well to basic supportive measures, and so many medical practitioners have successfully treated people with this syndrome, frequently without identifying the precise underlying cause [3,4].

## Case presentation

A 61-year-old male with a past medical history of diabetes mellitus, ESRD, hyperlipidemia, and hypertension presented to the emergency department for seizures at home. The patient had taken his premeal insulin but skipped his meal and had multiple seizures at home over 30 minutes after skipping his meal. The patient reported sweating and becoming lethargic and drowsy before the seizure. Blood sugar was found at 40 by the emergency medical services (EMS). He received midazolam 5 mg and dextrose 25 gm in the field, and on arrival at the emergency department (ED), he was still overwhelmed, and his blood sugar was still at 30. Another dextrose 50 injection was given, and a dextrose infusion was started. Home medications were reviewed, and of note was that the patient was on carvedilol 3.125 mg twice daily for more than one year for palpitations. Initial vitals showed a blood pressure of 100/60 mmHg and bradycardia with a heart rate of 50/min, followed by the sudden development of cardiac arrest. The return of spontaneous circulation was achieved in six minutes with cardiopulmonary resuscitation. The initial EKG just before cardiac arrest exhibited sinus bradycardia with first-degree heart block. After two doses of intravenous atropine, he was immediately started on a dopamine drip at 10 mcg/kg/min with a heart rate response of 50-55 bpm. He was placed on a cardiac monitor, and labs were drawn. He received fluids, and his dopamine drip was titrated to 15 mcg/kg/min. His labs on admission are illustrated in Table 1.

**Table 1:** Labs on admission.

Investigation	Value	Reference range
Hemoglobin	11.5	11.0-15.0 g/dL
Hematocrit	41.8	35-46%
White blood cell	8.9	3.8-5.3 × 10^6^/uL
Platelets	188	130-400 × 10^3^/uL
Glucose	223	80-115 mg/dL
Blood urea nitrogen	70	9.8-20.1 mg/dL
Creatinine	9.5	0.57-1.11 mg/dL
Sodium	130	136-145 mmol/L
Potassium	6.1	3.5-5.1 mmol/L
Chloride	101	98-107 mmol/L
Bicarbonate	21	23-31 mmol/L
Calcium	7.6	8.8-10.0 mg/dL
Albumin	3.7	3.2-4.6 g/dL
Magnesium	2.7	1.6-2.6 mg/dL
Brain natriuretic peptide (BNP)	392	10.0-100.0 pg/mL
COVID-19 polymerase chain reaction (PCR)	Positive	Negative
High-sensitivity troponin I	32	0.0-17.0 ng/L
Prothrombin time	9.9	9.8-13.4 s
International normalized ratio (INR)	0.83	0.85-1.15
Partial thromboplastin time (PTT)	41.5	24.9-35.9 s
Thyroid-stimulating hormone	1.154	0.465-4.680 uIU/mL
Free T4	0.81	0.78-2.19 ng/dL
Urine toxicology	Negative	Negative

The patient was COVID-19 positive on pre-admission screening for SARS-COVID-19. COVID markers are shown in Table 2. 

**Table 2:** COVID markers during hospitalization.

Investigation	Value	Reference value
Lactate dehydrogenase (LDH)	386	140-271 U/L
Procalcitonin	0.37	0.00-0.08 ng/mL
Erythrocyte sedimentation rate	80	0-20 mm/h
C-reactive protein	3.4	≤0.1 mg/L
D-dimer	1796	≤500 ng/mL DDU

**Figure 1:**
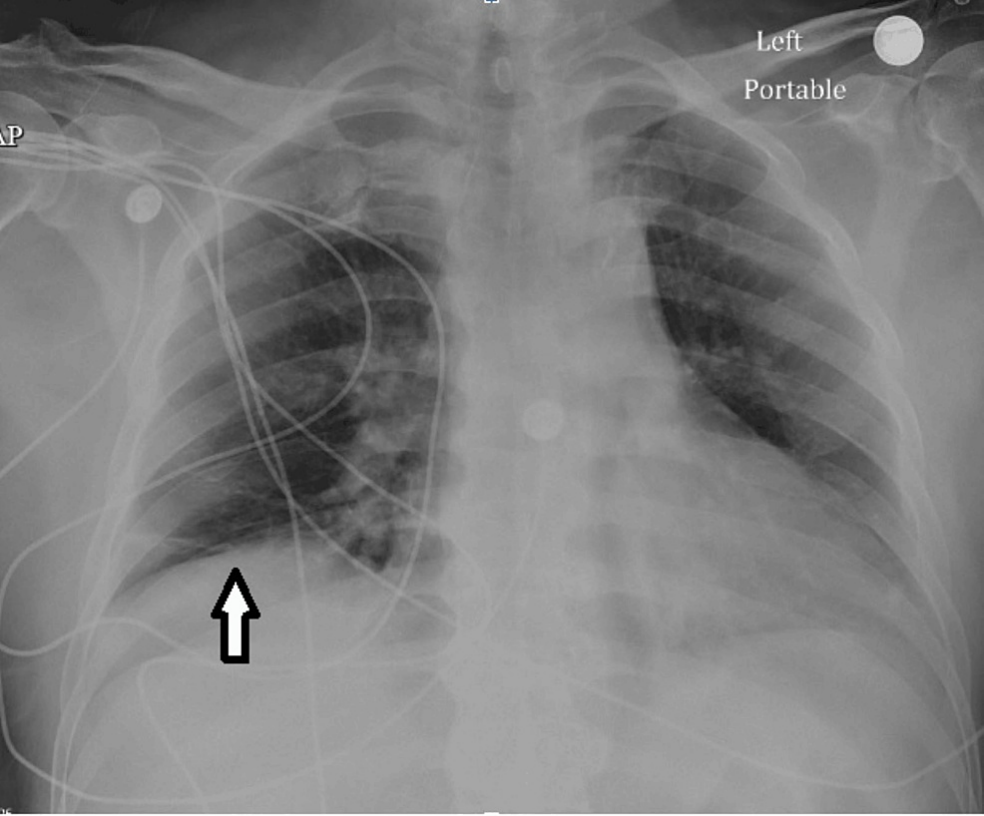
Chest X-ray on admission showing right basilar discoid atelectasis (white arrow).

**Figure 2:**
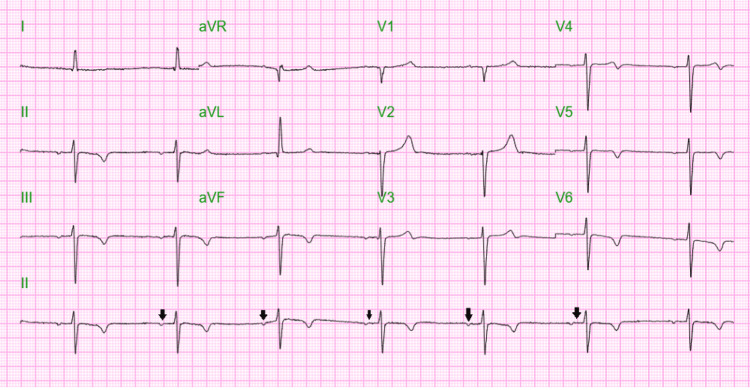
Electrocardiogram showed a heart block with junctional escape without any hyperkalemic changes (black arrows).

The chest X-ray showed right basilar discoid atelectasis, as shown in Figure 1. The EKG revealed a complete heart block with a junctional escape (Figure 2).

**Figure 3:**
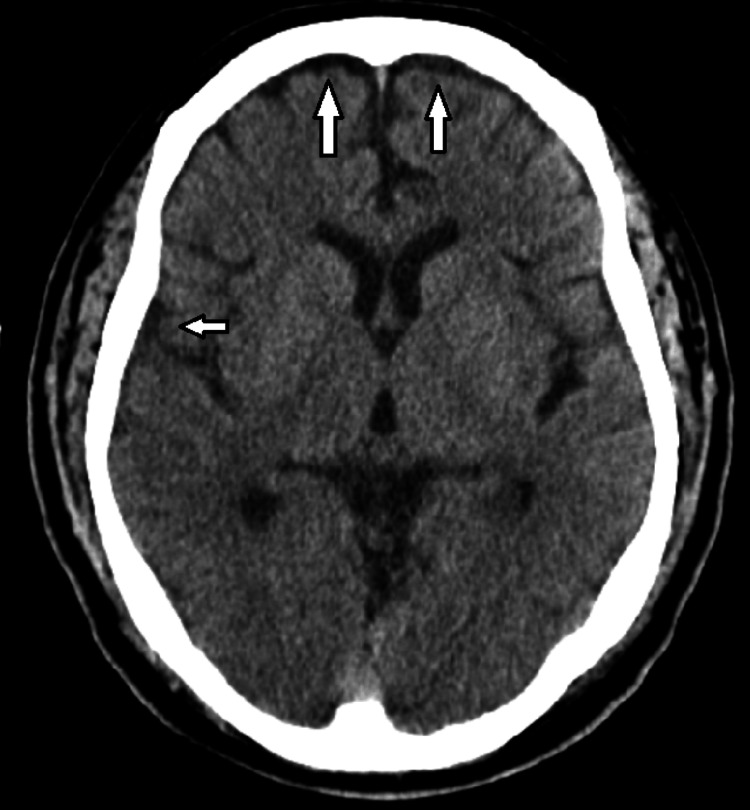
Computed tomography of the head showed mild diffuse chronic early senescent changes (white arrows).

Computed tomography of the head was performed on admission, showing mild diffuse chronic early senescent changes but no acute central nervous system pathology (Figure 3). 

**Figure 4:**
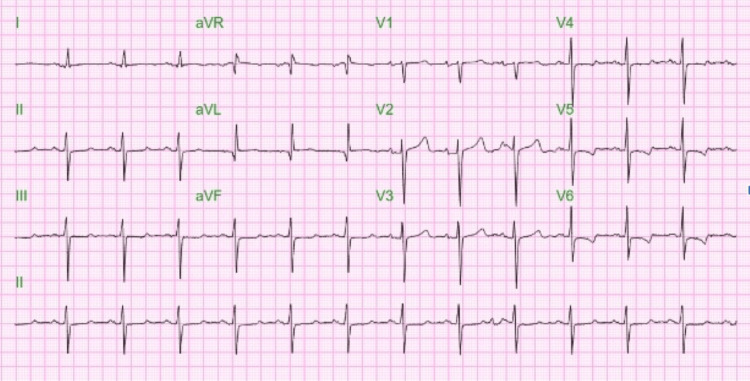
EKG after TVP placement showing normal HR at 77. HR: heart rate; TVP: transvenous pacemaker.

The patient has persistent bradycardia despite being treated with a dopamine drip at a maximal dose. For hyperkalemia, kayexalate (30 grams), intravenous calcium gluconate (two grams), albuterol nebulization, and intravenous regular insulin were given. Cardiology was consulted, and a temporary transvenous pacemaker (TVP) was planned in light of dopamine-resistant bradycardia and heart block. TVP was inserted through the right femoral approach. Following the procedure, hemodialysis was resumed through the right chest permacath. Permanent pacemaker placement (PPM) was deferred as the rhythm became regular, with a heart rate consistently between 70 and 80 beats per minute, and the telemetry monitoring revealed no pacing episodes. The EKG after 24 hours of TVP placement showed a heart rate of 77 beats per minute (Figure 4). Hyperkalemia improved with further dialysis sessions, and the temporary pacemaker was removed after three days.

## Discussion

The BRASH syndrome has a convoluted pathophysiology and clinical picture. While the underlying pathophysiology of BRASH syndrome has been poorly understood and known since the 1990s, the illness has recently been recognized as a distinct entity, and its real epidemiology is still largely unknown [1,5]. The initiation of the vicious cycle of BRASH syndrome follows several pathways. The most common triggers that have been found are dehydration and hypovolemia, acute kidney injury, the amplified effects of drugs, and generally any condition that promotes hyperkalemia or renal failure. 

Patients may exhibit a wide range of signs and symptoms, such as syncope, generalized weakness, dizziness/lightheadedness, altered mental status, dyspnea, and symptomatic bradycardia. This makes it extremely difficult to differentiate between patients presenting with any of the signs and symptoms of BRASH syndrome. To relate, this patient presented with bradycardia, hypoglycemia, and cardiac arrest [5]. 

Additionally, hyperkalemia itself is known to cause cardiac rhythm abnormalities such as bradycardia or even heart block, asystole, ventricular tachycardia, and ventricular fibrillation, especially at a potassium level of more than 7.0 mEq/L [2]. The patient described in this report presented with cardiac arrest and a complete heart block in the EKG with a potassium level of 7.1 mEq/L despite being on continuous renal replacement therapy. Additional EKG abnormalities, such as QRS widening and characteristic alterations to the P and T-wave morphology, are often observed in severe hyperkalemia but are rarely present in BRASH syndrome [6]. The BRASH syndrome occurs when the effect of hyperkalemia on AV node block is further exaggerated by synergistic entities like the accumulation of AV nodal blocking drugs like beta-blockers, calcium channel blockers, angiotensin-converting enzyme inhibitors, and angiotensin receptor blockers. COVID-19 infection has also been associated with AV nodal blockade although the precise mechanism of this is unclear [4]. Thus, in our case, we believe that hyperkalemia, along with the small dose of 3.125 mg BID of carvedilol, as well as the COVID-19 infection, might have compounded the AV nodal blockage, leading to the presentation of BRASH syndrome. Although research on the impact of viruses like COVID-19 on AV nodal blockage in BRASH syndrome patients is limited, clinicians should be mindful of the possibility. The development of BRASH syndrome in the context of end-stage renal disease (ESRD) and hyperkalemia in our patient was postulated to have occurred due to the direct myocardial damage and conduction block caused by the COVID-19 infection, which exaggerated the AV nodal blocking effects of hyperkalemia and beta-blocker accumulation. 

The mainstay of treatment for BRASH syndrome is fluids and vasopressors for the resuscitation and stabilization of vital functions, along with cautious management of concomitant conditions [5]. Stopping all AV nodal blocking agents and prompt hyperkalemia management are the first steps. Treatments to quickly lower serum potassium levels, such as cardiac membrane stabilization with IV calcium gluconate, and the administration of insulin with dextrose coupled with albuterol nebulization, should be immediately started. Patients with refractory cases would require emergency dialysis to improve their K+ levels. However, dialysis is sometimes difficult to perform in hemodynamically unstable patients, and the alternative of CRRT may not help to do away with the potassium fast enough [6]. Thus, in individuals experiencing bradycardia-induced shock, dopamine, epinephrine, or isoproterenol infusion are all acceptable modalities for corrective bradyarrhythmia-induced shock [1,7,8]. Procedures, such as transvenous pacing or hemodialysis, are often averted with aggressive medical treatment.

But there are instances when this isn't easy, like when the patient arrives late with cardiac arrest and every aggressive step has to be taken to improve the patient's outcome. One of the most typical mistakes made when managing BRASH syndrome is to treat it as a single entity instead of seeing it as a complicated ensemble [9,10]. Adverse outcomes and invasive management plans like implanting a permanent pacemaker can be avoided with early recognition and timely intervention. Better patient outcomes may be possible if the pathophysiology of BRASH syndrome could be better understood and recognized.

## Conclusions

BRASH syndrome in the setting of COVID-19 and ESRD is a rare but potentially life-threatening condition. Early recognition and prompt intervention, including correction of hyperkalemia, initiation of renal replacement therapy, and cardiac pacing, are essential for optimizing outcomes in these complex cases. Given the complex pathophysiology and high mortality risk, it is prudent to treat hyperkalemia aggressively medically, correct it, and avoid unnecessary invasive procedures. However, in cases where the patient is hemodynamically unstable, transvenous pacing may be necessary to improve the patient's prognosis. Overall, BRASH syndrome is an under-recognized entity, and more research is required to develop and implement efficient triaging methods, consistent diagnostic criteria, and therapeutic guidelines.
